# Benefits of Erinacines from Different Cultivate Formulas on Cognitive Deficits and Anxiety-Like Behaviour in Mice with Trimethyltin-Induced Toxicity

**DOI:** 10.21315/tlsr2023.34.3.9

**Published:** 2023-09-30

**Authors:** Yaovapa Aramsirirujiwet, Teerachart Leepasert, Danita Piamariya, Wachiryah Thong-asa

**Affiliations:** 1 Department of Microbiology, Faculty of Science, Kasetsart University, 50 Ngamwongwan Rd, Lat Yao, Chatuchak, Bangkok 10900, Thailand; 2 Department of Chemistry, Faculty of Science, Kasetsart University, 50 Ngamwongwan Rd, Lat Yao, Chatuchak, Bangkok 10900, Thailand; 3 Animal Toxicology and Physiology Specialty Research Unit (ATPSRU), Physiology Division, Department of Zoology, Faculty of Science, Kasetsart University, 50 Ngamwongwan Rd, Lat Yao, Chatuchak, Bangkok 10900, Thailand

**Keywords:** Erinacine, *Hericium erinaceus*, Hippocampus, Neurodegeneration, Trimethyltin

## Abstract

We investigated the neurological effects of the varied erinacine composition of different mycelia cultures in mice with trimethyltin (TMT)-induced neurodegeneration. Forty male ICR mice were randomly divided into five groups of Sham-veh, TMT-veh, TMT-EME, TMT-EMR and TMT-EME/R. The TMT groups received 2.6 mg/kg one-time intraperitoneal injections of TMT. Oral dosages of 200 mg/kg erinacine combination from each *Hericium erinaceus* mycelia (EM) cultivated formula (100% eucalyptus wood [E], 100% rubber wood [R], or 40% eucalyptus wood/60% rubber wood [E/R]) were given for two weeks. Spatial learning, memory, flexibility, and anxious behaviour were evaluated alongside brain tissues’ oxidative status and histological analyses. Erinacine composition from EME/R exhibited significant positive effects on spatial learning, memory, flexibility, and anxiety (*p* < 0.05). These findings emerged concurrently with the significant mitigation of hippocampal lipid peroxidation, CA1 hippocampal, cortical neuron, and corpus callosum white matter degeneration (*p* < 0.05). These neurological benefits were associated with the EME/R composition of erinacine A, C, D, G, H, I, K and R. The best neuroprotective effect against TMT-induced neurodegeneration in mice is offered by the EME/R erinacine composition according to its anti-lipid peroxidation, its nurturing effect on neuronal and white matter, and mitigation of behavioural deficits.

**Highlights**

- The best cultivate formula is EME/R.
- Erinacine composition from EME/R exhibited significant positive effects on spatial learning, memory, flexibility and anxiety.
- Neurological benefits are associated with the EME/R composition of erinacine A, C, D, G, H, I, K and R.

## INTRODUCTION

Neurodegenerative diseases are reported to have multiple pathomechanisms associated with neuronal and white matter damage with relevance to behavioural deficits. Damage to particularly vulnerable brain areas is uniquely correlated to neurodegenerative symptoms. In dementia (for instance, in Alzheimer’s disease), the vulnerable brain area affected is that of the limbic structures, especially the hippocampus *(*Hock *et al*. 2000; Lee *et al*. 1999; Mattila *et al*. 1999; Sze *et al*. 1997). This area plays a major role in emotional and cognitive skills, such as learning and memory (Dillon *et al*. 2008; Li *et al*. 2011; Shi *et al*. 2006; Silvers *et al*. 2003; Win-Shwe *et al*. 2008). Similarities between dementia symptoms (i.e., declines in emotional and cognitive capacities) are due to their origin in the same vulnerable brain area that is affected. Multiple pathomechanism (i.e., mitochondrial and endothelial dysfunction, oxidative stress and inflammation, alteration of neurotransmission and synaptic signaling) has been reported for neuronal and white matter degenerative processes with concurrent therapeutic targets (Butterfield *et al*. 2001; Chong *et al*. 2005). Induced by the organotin compound trimethyltin chloride (TMT), neurodegenerative phenomena have been reported to share crucial pathogenic pathways common to most neurodegenerative diseases (Lee *et al*. 2016). TMT-induced neurotoxicity is regarded as a useful tool in providing an animal model of neurodegenerative disease, advantaging cognitive impairment with selectively neuronal damage (Geloso *et al*. 2011). The molecular pathogenesis of TMT is composed of complex events, including glutamate excitotoxicity, impairment of neuronal transmission, intracellular calcium augmentation, oxidative stress, and apoptotic and necrotic cell death (Ogita *et al*. 2004; Wang *et al*. 2008; Yoneyama *et al*. 2009). Neuronal degeneration is selectively localised in the limbic structures, in particular the hippocampus; therefore, TMT also causes cognitive behavioural deficits correlated with selectively neuronal structural damage.

For a long period of time, nutraceutical and alternative medicine in Asian countries has used erinacine-enriched *Hericium erinaceus* mycelia for its beneficial properties for human health. The beneficial effects of *H. erinaceus* are antimicrobial, anticancer, antihyperglycemic, antilipidemic, immune nurturing, antidepressant and antioxidant (Chiu *et al*. 2018; Hiwatashi *et al*. 2010; Li *et al*. 2014; Wang *et al*. 2005). The fermented mycelia of *H. erinaceus* have been reported to produce a variety of bioactive compounds. The most abundant terpenoids from cultured mycelia, erinacines, have been found to be neuroprotective against neurodegenerative diseases such as ischemic stroke and Alzheimer’s and Parkinson’s diseases (Li *et al*. 2018). The erinacines group of cyathin diterpenoids is composed of 15 members (erinacine A–K, and P–S), some of which (A–I) are reported to have various neurohealth properties (Li *et al*. 2018). The neurohealth effect of erinacines is due to its low molecular weight, which easily crosses the blood brain barrier. Most erinacines act as potent nerve growth factor (NGF) stimulators (e.g., erinacine A–F and H) which benefit neurohealth by preventing neuronal death, promoting neurite outgrowth, and maintaining neuronal functions. Variety of benefits are also indicated, i.e., activation of brain derived neurotrophic factor (BDNF), promote neuron growth and regeneration, modulation of neurotransmitters, neuronal apoptotic mitigation, anti-oxidation, anti-inflammation, modulation of neuropathic pain, and reduction of amyloid plaque in Alzheimer’s animal model (Kawagishi *et al*. 1994; Lee *et al*. 2000; Ma *et al*. 2010).

Biosynthesised erinacines are highly desirable due to the complexity of chemical syntheses, which are multistep processes with low yield and purity. The use of a different cultivation formula can alter the yield of erinacine composition. The *H. erinaceus* mycelia used in the present study were produced by different cultivation formulas, resulting in different compositions of erinacines. We expected erinacines from the best cultivation formula to provide neuroprotection against the TMT-induced neurodegenerative disease of our mice model. Therefore, the present study aimed to evaluate the neuroprotective effects of the erinacine composition produced by different *H. erinaceus* mycelia cultivation formulas in mice with TMT-induced neurodegeneration.

## MATERIALS AND METHODS

### Chemicals and Reagents

Trimethyltin chloride, dimethyl sulfoxide (DMSO), paraformaldehyde (PFA), malondialdehyde (MDA), hydrogen peroxide, superoxide dismutase (SOD), sodium dodecyl sulfate, trichloroacetic acid, epinephrine, ethylenediamine tetraacetic acid (EDTA), ethanol, xylene, cresyl violet and luxol fast blue were purchased from Merck, Millipore.

### Animals

Forty 8-week-old male ICR mice (*Mus musculus*) weighing 30 g–50 g were obtained from National Laboratory Animal Centre, Mahidol University, Salaya, Nakhon Pathom Province, Thailand. All mice were housed one per cage and allowed to habituate in a room with controlled temperature (25°C) and humidity (55%) for 4 weeks before the experiment began.

### EM Characterisation

Three different cultivation formulas: (i) EME (100% eucalyptus wood [E]), (ii) EMR (100% rubber wood [R], and (iii) EME/R (40% eucalyptus wood and 60% rubber wood [E/R]) were chosen to produce erinacine-enriched mycelia (EM). *H. erinaceus* mycelia were cultivated (substrate composed of corn cob [15 g]), rice bran [20 g]), sawdust [65 g]), moisture 50%–60% and pH 3.6–6.6) and delivered by our co-authors in the Department of Microbiology, Faculty of Science, Kasetsart University; analysis of erinacine content was performed with high performance liquid chromatography mass spectrometry (HPLC-MS) by our co-author in the Department of Chemistry, Faculty of Science, Kasetsart University. All details were described in our previous report (Darmasiwi *et al*. 2022).

### Experimental Protocol

**Figure 1:**
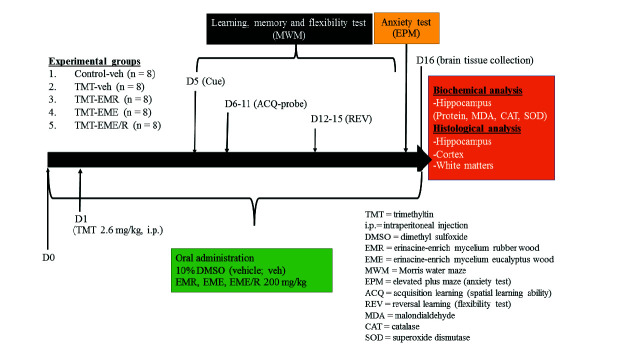
The experimental protocol.

The experimental protocol and animal care were approved by the Animal Ethics Committee, Faculty of Science, Kasetsart University (ID#ACKU63-SCI-004). Mice were randomly divided into 5 groups: (i) Sham-veh (*n* = 8), (ii) TMT-veh (*n* = 8), (iii) TMT-EME (*n* = 8), (iv) TMT-EMR (*n* = 8) and (v) TMT-EME/R (*n* = 8). Sham-veh was a control group that receive similar procedure as the other group and receive only vehicle (veh). Neurodegeneration was induced in the TMT groups by one-time intraperitoneal injection of 2.6 mg/kg of TMT which the dose is able to induce neurological changes and cognitive behavioural deficits in mice (Thong-Asa *et al*. 2020). Erinacines from different *H. erinaceus* mycelia cultivated formula named EME, EMR and EME/R were orally given 24 h prior to TMT injection, and continued for 2 weeks with a dosage of 200 mg/kg in 10% DMSO (veh). We selected EM dose as 200 mg/kg according to a previous study of *H. erinaceus* mycelium, and has been proved as the lowest active dose that exhibited neuroprotective effect with anti-inflammatory properties (Chiu *et al*. 2018). Animal’s health was routinely assessment, i.e., checking of weight, food intake and ordinary behavior. If animal’s weight reduced more than 20% or loss of motor activity, they were excluded. Behavioural tests were conducted to evaluate sensorimotor (cue test), spatial learning (ACQ), memory (ACQ probe), learning flexibility (REV) and anxiety-like behaviour (EPM). After that, the mice were killed and brain tissue was collected for the analysis of hippocampal oxidative status, neuronal and white matter histology (Fig. 1).

### Sensorimotor Evaluation

Sensorimotor evaluation was use for evaluation of visual and swimming abilities of each mouse and conducted as a cue test. This test was used to evaluate visual and swimming abilities before the spatial cognition tests in the Morris water maze (MWM). The MWM consisted of a 150-cm diameter circular pool with a centrally located visible platform. All mice were allowed to find the platform for 4 trials (within a time limit of 120 sec/trial) with 30 sec inter-trial intervals. A trial ended when the mouse climbed onto the platform. The swim speed (cm/sec) was recorded and used for sensorimotor evaluation. All MWM test was perform in environmental controlled condition, same time test during 6.00 p.m. to 12.00 p.m., with constant 200 lux illumination (above water surface) and quiet background noise (Manyagasa & Thong-asa 2019).

### Acquisition Test for Spatial Learning Ability Evaluation

The day after the cue test, spatial learning ability was evaluated for five consecutive days (D1–D5) by acquisition (ACQ) test sessions. The platform was submerged 2 cm below the water’s surface in the selected target quadrant. All mice were given 4 trials/day with 30 sec inter-trial intervals. The escape latencies from D1–D5 of the ACQ sessions were analysed for spatial learning ability (Manyagasa & Thongasa 2019).

### Acquisition Probe for Spatial Memory Capacity Evaluation

When the ACQ session was done, an ACQ probe test was delivered to assess spatial memory capacity. The hidden platform was removed and mice were allowed to search the platform for 60 sec. The percentage of time spent in the target quadrant was analysed and interpreted as spatial memory capacity (Manyagasa & Thong-asa 2019).

### Reversal Test for Learning Flexibility Evaluation

Learning flexibility was evaluated in the MWM for three consecutive days (D6–D8) after the ACQ probe test, in a process named the reversal (REV) session. All procedures were similar to those of the ACQ sessions, but the hidden platform was switched to the opposite quadrant (Manyagasa & Thong-asa 2019). All the MWM experiments were recorded and analysed using Smart© version 3.0.04 (Harvard Apparatus).

### Elevated Plus Maze Test for Anxiety-Like Behaviour

The elevated plus maze (EPM) test was carried out after completion of the MWM tests. The EPM was a black, acrylic, plus-shaped maze elevated 50 cm above the ground. It comprised two opposing closed arms and two opposing open arms with a central junctional area. Each mouse was placed in the centre, facing one of the open arms, and given 5 min to freely explore the maze; the time spent in each type of arm was recorded. Anxiety-like behaviour was indicated by the percentage of time spent in the open and closed arms (Manyagasa & Thong-asa 2019).

### Biochemical Evaluation of Hippocampal Oxidative Status

All mice were euthanised via intraperitoneal injection of more than 60 mg/kg sodium pentothal. They were decapitated, the brains were quickly removed, and the hippocampi were collected, washed in a cool 0.9% normal saline solution, and homogenised in potassium-phosphate buffer (0.05 M, pH 7.4). The homogenates were kept and divided into two parts; the first part was used to estimate the lipid peroxidation product MDA. In brief, we mixed 0.2 mL of brain homogenate with 4% sodium dodecyl sulfate, 1.5 mL of 20% acetic acid and 1.5 mL of 0.5% thiobarbituric acid, heated the mixture at 95°C for 1 h. and then centrifuged it for 10 min at 3,500 rpm before reading the supernatant at 532 nm. Calculation of MDA concentration using the standard curve of MDA concentration 0, 0.93, 1.85, 2.76, 3.66 and 4.55 μM (y = 0.148x – 0.1082; R^2^ = 0.9973) and presented as μM/mg of protein. The second part of homogenate was centrifuged at 10,000 ×g at 4°C for 10 min and supernatant was used to evaluate SOD and catalase (CAT) activity (Sakamula & Thong-asa 2018).

### Histological Analysis

Brains were kept in 4% PFA for 24 h before being processed, embedded in paraffin, and cut into 5-μm sections. Five pieces of brain sections from each animal were selected starting from −1.94 mm of the bregma, which covered the specific brain area of interest (Paxinos & Franklin 2008). In order to avoid counting the same neuron in each brain area, each section was selected from a space interval of 125 μm (Thong-Asa & Bullangpoti 2020; Thong-Asa *et al*. 2017).

Brain slides were stained with a standard protocol of 0.1% cresyl violet and 0.1% luxol fast blue, for neuronal and myelinated fibre identification, respectively (Somredngan & Thong-asa 2017). After the staining process, specific brain areas were captured, including corpus callosum (CC) white matter, the cortical projecting neurons, the hippocampal subregions cornus ammonis 1 (CA1) and cornus ammonis 3 (CA3), and the dentate gyrus (DG). With 200x magnification, used 3 non-overlapping images from both hemispheres for neuronal counting using Image J. Viable cells were characterised as light purple and a clear nucleus and nucleolus. In contrast, cells that had undergone shrinkage were dark purple with a barely visible nucleus and nucleolus and defined as degenerated. We interpreted neuronal data as % degeneration using the formula:


$$\%\;\text{degeneration}=\frac{\text{degenerated}}{\text{degenerated}+\text{viable}}\times 100$$

We captured white matter in the CC at 200× magnification and analysed three non-overlapping images from both hemispheres using Image J. We presented the data from the intact fiber with luxol fast blue (LFB) as % area of white matter intact. The myelinated fiber density of CC white matter and number of viable and degenerating neurons in the cortex and hippocampus were blind assessed by two investigators using the National Institutes of Health (NIH) Image J.

### Statistical Analysis

Statistical analysis was performed with StatView for Windows, SAS institute Inc. (StatView version 5.0, SAS Campus Drive, Cary NC). The data were represented as mean ± standard error of mean (S.E.M.). Shapiro–Wilk test was employed to test the normality of data distribution. Escape latencies from ACQ and REV sessions were analysed by repeated-measured analysis of variance (ANOVA) and Fisher’s PLSD post hoc test. The swim speed of cue test, % time spent in the target quadrant of the ACQ probe, % time spent in the open and closed arms of the EPM test, and biochemical and histological data were analysed by one-way ANOVA and Fisher’s PLSD post hoc test. Statistical significance was accepted when *p*-value fell below 0.05.

## RESULTS

### Erinacine Characterisation

**Figure 2:**
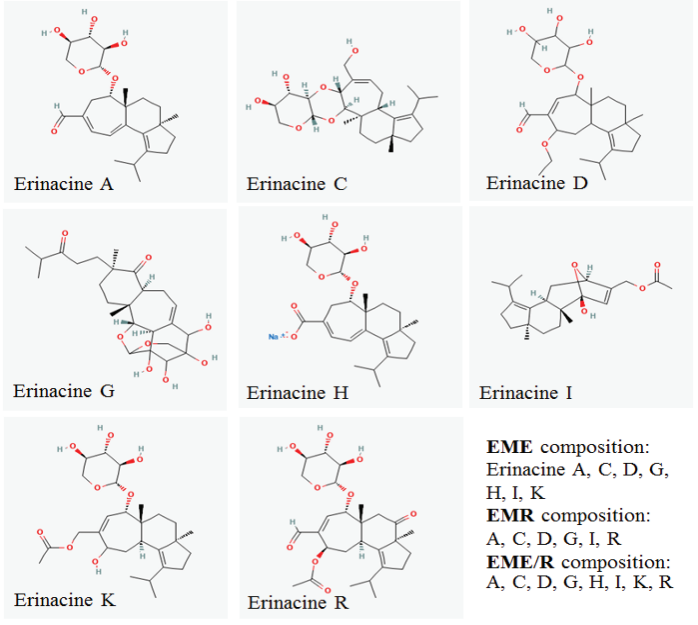
Erinacine composition of each cultivation formula (Kim *et al*. 2020).

Characterisation of erinacines from each cultivate using HPLC-MS indicated that EME was composed of erinacines A, C, D, G, H, I and K; EMR was composed of erinacines A, C, D, G, I and R; and EME/R was composed of erinacines A, C, D, G, H, I, K and R (Fig. 2).

### Sensorimotor Evaluation

Sensorimotor evaluation in the cue test showed no effect of TMT or treatments on the swim speed of all groups. This indicated equal visual and motor abilities in the MWM (Table 1).

### Spatial Cognitive Abilities

**Figure 3:**
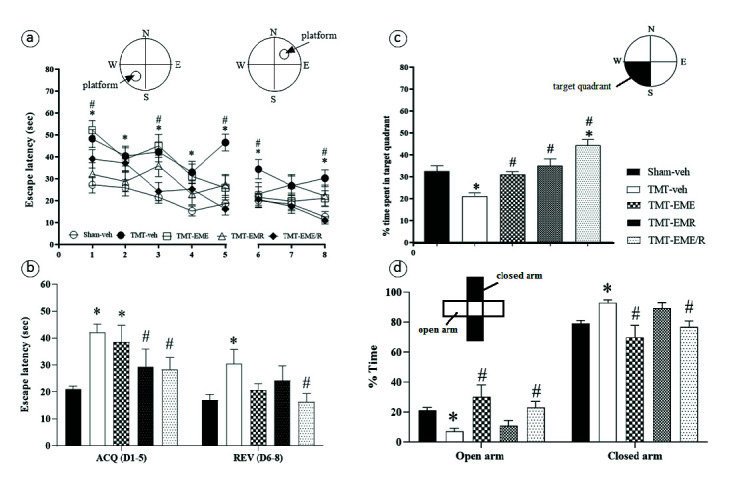
The elements of spatial learning evaluated by the MWM are (a–b) spatial acquisition and flexibility and (c) memory capacity; the black colour represents the target quadrant. (d) Histograms of % time in the open and closed arm in elevated plus maze. *Indicates significant difference compared to Sham-veh. ^#^Indicates significant difference compared to TMT-veh (*n* = 8 for each group).

The spatial learning ability assessed by the ACQ test showed a TMT-induced spatial learning deficit when compared to that of the Sham-veh group (*p* = 0.0009). Only EMR (*p* = 0.0455) and EME/R (*p* = 0.0208) depicted significant improvement of the spatial learning deficit (Figs. 3a and 3b). The spatial memory capacity tested in the ACQ probe revealed that TMT induced a significant memory deficit unexperienced by the Sham-veh group (*p* = 0.0003). All of the EME, EMR and EME/R treatments significantly improved spatial memory deficit, unlike the untreated TMT-veh group (*p* = 0.0124, *p* = 0.0002 and *p* < 0.0001, respectively). The REV test of learning flexibility showed that TMT significantly induced flexibility deficit beyond the sham-veh baseline (*p* = 0.0131) and that EME/R significantly reversed the deficit level seen in the TMT-veh group (*p* = 0.0114). Interestingly, EME/R significantly enhanced spatial memory capacity beyond the sham-veh level (*p* = 0.0006; Fig. 3c).

### Anxiety-like Behaviour

In Fig. 3d, anxiety-like behaviour in the EPM test resulted in a significant increase in anxiety 2 weeks after TMT induction compared to that of the Sham-veh group (*p* = 0.0089). Only EME- and EME/R-treated mice showed significant anxiolytic behaviour, represented by their significantly greater time spent in the open arm when compared to TMT-veh (*p* = 0.0006 and *p* = 0.0039, respectively).

### Oxidative Status

Trimethyltin exhibited significant induction of hippocampal lipid peroxidation (Table 1). This indicated by significant increased MDA level when compared TMT-veh to Sham-veh (*p* = 0.0023). In addition, the hippocampal oxidative status assessment revealed significant differences only between the TMT-veh and TMT-EME/R groups (*p* = 0.0319). This result indicated benefit of erinacine composition from EME/R against hippocampal lipid peroxidation that was induced by TMT. In addition, EME/R seems to have enhanced SOD activity, the result indicated the increase level of this enzymatic activity higher than Sham-veh and TMT-veh, though this enhancement is not statistically (*p* = 0.0692 and *p* = 0.0574, respectively).

### Histology

**Figure 4:**
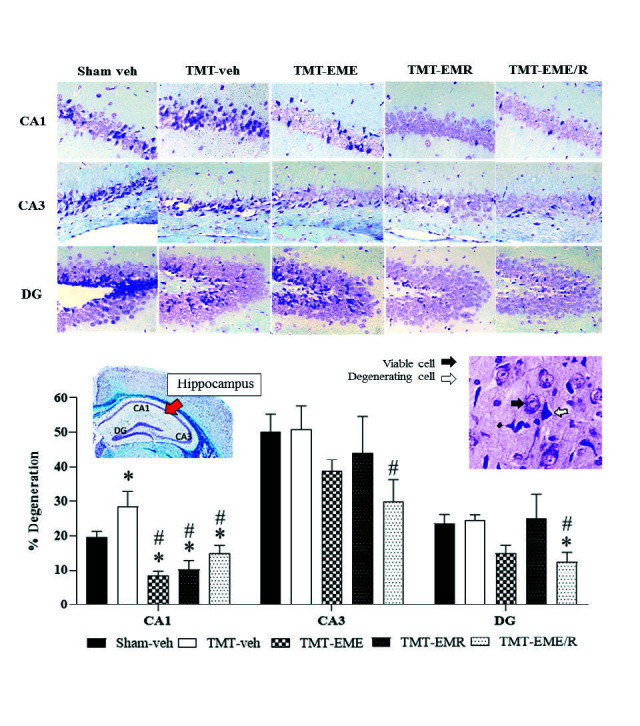
Representative photomicrograph of hippocampus CA1, CA3 and DG neurons stained with 0.1% cresyl violet at 200× magnification. Red arrow indicates the hippocampal area of interest. Black and white arrows represent viable and degenerating cells, respectively. The histogram showed the % degeneration of neurons in CA1, CA3 and DG. *Indicates significant difference compared to Sham-veh; ^#^Indicates significant difference compared to TMT-veh (*n* = 4 for each group).

**Figure 5:**
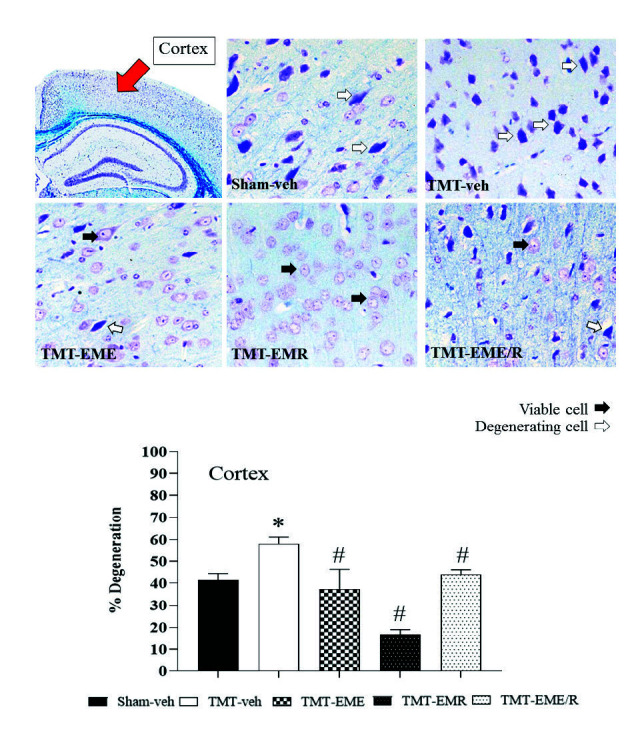
Representative photomicrograph of cortical projecting neurons stained with 0.1% cresyl violet at 200× magnification. Red arrow indicates the cortical area of interest. Black and white arrows represent viable and degenerating cells, respectively. The histogram showed the % degeneration of cortex neurons. *Indicates significant difference from the Sham-veh group; ^#^Indicates significant difference from the TMT-veh group (*n* = 4 for each group).

**Figure 6:**
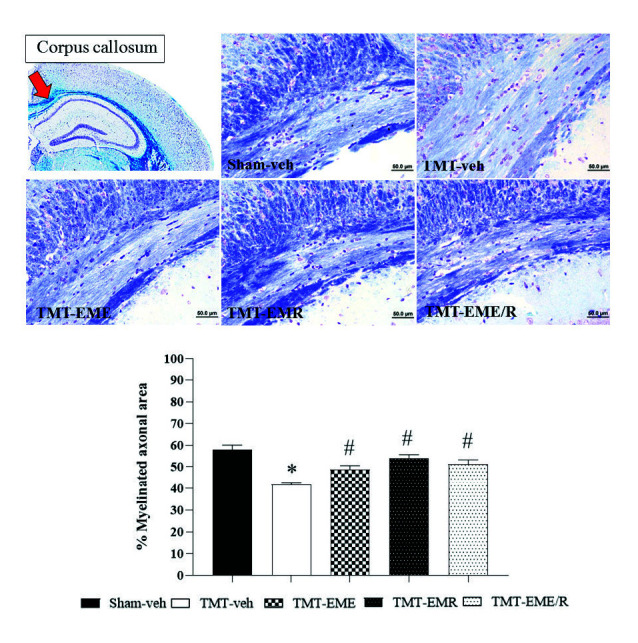
Representative photomicrograph of CC white matter stained with 0.1% luxol fast blue at 200× magnification, scale bar = 50 μm. Red arrow indicates the area of CC. The histogram showed the % myelinated axonal area of the CC. *indicates significant difference from the Sham-veh group; ^#^Indicates significant difference from the TMT-veh group (*n* = 4 for each group).

Brain histological evaluation revealed significantly greater neuron degeneration in the cortex (Fig. 5, *p* = 0.0038) and CA1 hippocampal area (Fig. 4, *p* = 0.033) of TMT treatment group mice than in that of the Sham-veh mice. White matter density in the CC revealed the same result: that TMT induced a significant reduction in CC myelinated fiber density (Fig. 6, *p* < 0.0001). These results demonstrate that TMT selectively damaged not only the hippocampus, but also the cortex neurons and CC white matter.

Regarding the hippocampal subregions, CA1 (but not CA3 or DG) was significantly damaged by TMT induction; however, EME, EMR and EME/R treatment significantly mitigated CA1 degeneration (*p* < 0.0001, *p* < 0.0001 and *p* < 0.0015, respectively). In CA3 and the DG, though TMT had not significantly induced neuronal degeneration, EME/R treated mice showed significant decreases in degenerating cells compared to those of the TMT-veh mice (*p* = 0.0342 and *p* = 0.0157, respectively). Interestingly, EME/R significantly reduced degenerating cells beyond Sham-veh levels (Fig. 4, *p* = 0.0341), which indicated some effect of EME/R on specific neurogenesis brain areas.

The cortical projecting neurons and CC white matter density were similar across all EM-treated mice. Those treated with EME, EMR, and EME/R showed significantly slowed cortex cell degeneration (Fig. 5, *p* = 0.0013, *p* < 0.0001 and *p* < 0.0184, respectively) and the reduction of myelinated fibre in the CC (Fig. 6, *p* = 0.0036, *p* < 0.0001 and *p* < 0.0001, respectively).

## DISCUSSION

The present study demonstrates the neuroprotective effect of erinacines from *H. erinaceus* mycelia in mice with TMT-induced neurodegeneration. We found that erinacines from all EM could prevent the degeneration of neurons in the cortex, CA1 hippocampus, and CC white matter, with precise behavioural correlation. Significant deficits in the spatial cognitive behaviors assessed in the MWM (including spatial learning, memory and flexibility) were apparent 2 weeks after TMT induction. Only the EME/R treatment group exhibited significant improvement in these all aspects, which implies that EME/R prevents neuronal and white matter degeneration and is associated with functional restoration. EMR improved spatial learning and memory but not flexibility, while EME improved only spatial memory and neither spatial learning nor flexibility. This divergence is due to differences in erinacine composition. The HPLC-MS results demonstrated that EME/R has the most type of erinacines, including A, C, D, G, H, I, K and R. The erinacine composition of EME includes A, C, D, G, H, I and K, while EMR has erinacines A, C, D, G, I and R. From these different compositions emerges a group of erinacines (A, C, D, G and I) that is commonly found in all EM enough for ameliorating the degeneration of the cortex, CC white matter, and CA1 that is correlated with spatial memory impairment. Reported evidence shows that erinacines A–I exhibit various neurohealth properties (Li *et al*. 2018). Most of these erinacines act as potent nerve growth factor (NGF) stimulators (erinacines A–F and H), which prevent neuronal death, promote neurite outgrowth, and maintain neuronal functions (Kawagishi *et al*. 1994; Lee *et al*. 2000; Ma *et al*. 2010). Though all of the EM exhibited neuroprotective effects against neuronal and white matter damage, EME did not improve cognitive function (e.g., acquisition of spatial learning) and we notice the absence of erinacine R. It is interesting that, at present, no study of erinacine R has been conducted. Neither EME nor EMR visibly improved learning flexibility. We proposed that the combination of erinacines R, H and K would have some effect on learning flexibility, but this is refuted by the fact that neither EME (which has erinacines H and K, but not R) nor EMR (which has erinacine R, but not H or K) affect the restoration of learning flexibility. According to biochemical analysis result, only EME/R exhibited significant mitigation effect on hippocampal MDA increment which indicated notable effect of erinacine combination of EME/R in anti-lipid peroxidation. Therefore, non-common erinacines found in EME/R also participate in nurturing effect against neuronal oxidation. In addition, the present study observed neuronal histology in only the cortex and hippocampus, therefore, it cannot account for other brain areas that are responsible for cognitive flexibility, the neural basis of which involves many of the cortical regions, the orbitofrontal cortex, the striatum, the amygdala and the hippocampus (Izquierdo *et al*. 2016). Assessment of surviving or degenerating cells cannot be illustrate all aspects of cellular functioning. Therefore, aid of further study with cellular physiology and metabolism are needed.

Spatial learning and memory are hippocampal-dependent types of cognition (Dillon *et al*. 2008; Holahan & Routtenberg 2011; Li *et al*. 2011). Impairment of spatial learning and memory is particularly associated with CA1 and CA3 neurodegeneration (Dillon *et al*. 2008; Holahan & Routtenberg 2011). In the present study, spatial learning and memory impairments are associated with degeneration in CA1 but not in CA3. In addition, our results demonstrate that TMT selectively damaged not only the CA1 hippocampus, but also the cortex neurons and CC white matter. This can be explained by the CA1 subregion’s sensitivity and responsibility for spatial learning and memory. The evidence revealed the effect of TMT on CA vulnerability according to dose, duration, age, and animal strain differences (Geloso *et al*. 2011). Although CA3 and the DG were not significantly damaged by TMT, EME/R-treated mice showed enhancement of these two areas through significant decreases in degenerating cells. This may involve the activating effect of erinacine-enriched *H. erinaceus* on NGF and brain-derived neurotrophic factor. These two factors play important roles in neuronal strength and survival in pathological conditions. Moreover, brain-derived neurotrophic factor contributes to dendritic plasticity and hippocampal neurogenesis, especially in the DG subregion (Chiu *et al*. 2018). Our results indicated some effect of EME/R on specific neurogenesis brain areas.

The present study demonstrated that TMT significantly induced anxiety. This is because TMT can cause neurodegeneration in anxiety-related brain areas, for instance, the hippocampus and amygdala (Geloso *et al*. 2011). We also observed the anxiolytic effect of EME and EME/R. These treatments’ similar erinacine compositions included erinacines A, C, D, G, H, I and K, while EMR did not exhibit anxiolytic effects and lacked erinacines H and K. We suggested that erinacines H and K might have some effect on anxiety-like behaviour. Evidence of the antidepressant and antianxiety effect of erinacine-enriched *H. erinaceus* was demonstrated, suggesting NGF involvement (Nagano *et al*. 2010). Recently, molecular mechanisms of erinacine-enriched *H. erinaceus* have been elucidated. Erinacine-enriched *H. erinaceus* affects the modulation of neurotransmitters (e.g., norepinephrine, dopamine, and serotonin) and proinflammatory cytokines (e.g., interleukin-6 and tumour necrosis factor-alpha) (Chiu *et al*. 2018). The unknown potential benefit of erinacine combinations such as erinacines H, K, and R is very interesting. To date, erinacine H’s benefit in NGF activation has been clearly reported (Lee *et al*. 2000), but the effects of erinacines K and R have still not been studied especially in the aspect of neurohealth benefit. These prospective erinacine compositions may illuminate our further study and acquisition of valuable data on the best neuroprotection offered by erinacine composition. The present study observed neuronal histological change only in the hippocampus, but it may be that other affected brain areas (for instance, subregions of the amygdaloid complex) contribute to anxiety (Martin *et al*. 2009).

The present study examined only two weeks after TMT 2.6 mg/kg one-time intraperitoneal injection in mice, finding that TMT did not significantly change oxidative parameters represented by CAT, and SOD levels. It has been reported that TMT exhibits more acute toxic effects in mice immediately after exposure (Geloso *et al*. 2011); therefore, two weeks after TMT injection, the effect on oxidative stress was minimised. However, we indicated significant change of MDA level in the hippocampal area. A study also indicated significance increase of MDA level after TMT injection in mice (Jeong *et al*. 2021). This indicated the lipid peroxidation activation after TMT exposure in this specific brain area, and the mice treated with EME/R exhibited significantly reduced MDA levels. This indicated benefit of erinacine composition from EME/R against hippocampal lipid peroxidation that was induced by TMT. Research has reported that *H. erinaceus* treatment can prevent the increase of MDA (Durmus *et al*. 2021). Although we did not observe oxidative status in other brain areas such as cortex or striatum that can be related to behavioural tests, we found significant damage in cortex and white matter as well. In addition, the complex events of TMT-induced neurodegeneration, rather than oxidative stress, have been described. TMT pathomechanisms include calcium augmentation (overload) from excitotoxicity, neuroinflammation, mitochondrial damage and alteration of neurotransmitters (Geloso *et al*. 2011). Therefore, the benefit of the variety of erinacines in each EM is considered to aid the neuronal nurturing of both structure and function against multiple pathogenic mechanisms.

## CONCLUSION

We conclude that the best neuroprotective effect against TMT-induced neurodegeneration in mice of erinacine composition is produced by EME/R. Erinacine composition from EME/R exhibited significant positive effects on spatial learning, memory, flexibility and anxiety. The specific advantages of EME/R are its anti-lipid peroxidation, its improvement of neuronal and white matter, and its mitigation of behavioural deficits.

## Figures and Tables

**Table 1:** Sensorimotor and biochemical analysis of the hippocampal oxidative status (*n* = 4 for each group). Data represented as mean±S.E.M.

Parameters	Sham-veh	TMT-veh	TMT-EME	TMT-EMR	TMT-EME/R
Swim speed (cm/sec)	16.28 ± 0.79	14.46 ± 0.66	14.55 ± 0.73	14.20 ± 1.05	14.36 ± 0.67
MDA (μM/mg protein)	0.110 ± 0.04	0.132 ± 0.033*	0.120 ± 0.032	0.123 ± 0.001	0.038 ± 0.017#
CAT (U/mg protein)	0.106 ± 0.003	0.086 ± 0.016	0.095 ± 0.005	0.096 ± 0.006	0.108 ± 0.005
SOD (U/mg protein)	0.049 ± 0.016	0.026 ± 0.008	0.045 ± 0.015	0.042 ± 0.007	0.067 ± 0.009

*Notes*:

* Indicates significant difference from the Sham-veh group.

# Indicates significant difference from the TMT-veh group.

## References

[b1-tlsr-34-3-165] Butterfield DA, Howard BJ, LaFontaine MA (2001). Brain oxidative stress in animal models of accelerated aging and the age-related neurodegenerative disorders, Alzheimer’s disease and Huntington’s disease. Current Medicinal Chemistry.

[b2-tlsr-34-3-165] Chiu C-H, Chyau C-C, Chen C-C, Lee L-Y, Chen W-P, Liu J-L, Lin W-H, Mong M-C (2018). Erinacine A-Enriched *Hericium erinaceus* Mycelium produces antidepressant-like effects through modulating BDNF/PI3K/Akt/GSK-3β signaling in mice. International Journal of Molecular Sciences.

[b3-tlsr-34-3-165] Chong ZZ, Li F, Maiese K (2005). Oxidative stress in the brain: Novel cellular targets that govern survival during neurodegenerative disease. Progress in Neurobiology.

[b4-tlsr-34-3-165] Darmasiwi S, Aramsirirujiwet Y, Kimkong I (2022). Biological activities and chemical profile of *Hericium erinaceus* mycelium cultivated on mixed red and white jasmine rice. Food Science and Technology.

[b5-tlsr-34-3-165] Dillon GM, Qu X, Marcus JN, Dodart JC (2008). Excitotoxic lesions restricted to the dorsal CA1 field of the hippocampus impair spatial memory and extinction learning in C57BL/6 mice. Neurobiology of Learning and Memory.

[b6-tlsr-34-3-165] Durmus A, Durmus I, Bender O, Karatepe O (2021). The effect of *Hericium erinaceum* on the prevention of chemically induced experimental colitis in rats. Korean Journal of Internal Medicine.

[b7-tlsr-34-3-165] Geloso MC, Corvino V, Michetti F (2011). Trimethyltin-induced hippocampal degeneration as a tool to investigate neurodegenerative processes. Neurochemistry International.

[b8-tlsr-34-3-165] Hiwatashi K, Kosaka Y, Suzuki N, Hata K, Mukaiyama T, Sakamoto K, Shirakawa H, Komai M (2010). Yamabushitake mushroom (*Hericium erinaceus*) improved lipid metabolism in mice fed a high-fat diet. Bioscience, Biotechnology, and Biochemistry.

[b9-tlsr-34-3-165] Hock C, Heese K, Hulette C, Rosenberg C, Otten U (2000). Region-specific neurotrophin imbalances in Alzheimer disease: Decreased levels of brain-derived neurotrophic factor and increased levels of nerve growth factor in hippocampus and cortical areas. Arch Neurology.

[b10-tlsr-34-3-165] Holahan MR, Routtenberg A (2011). Lidocaine injections targeting CA3 hippocampus impair long-term spatial memory and prevent learning-induced mossy fiber remodeling. Hippocampus.

[b11-tlsr-34-3-165] Izquierdo A, Brigman JL, Radke AK, Rudebeck PH, Holmes A (2016). The neural basis of reversal learning: An updated perspective. Neuroscience.

[b12-tlsr-34-3-165] Jeong ES, Bajgai J, You IS, Rahman MH, Fadriquela A, Sharma S, Kwon HU, Lee SY, Kim CS, Lee KJ (2021). Therapeutic effects of hydrogen gas inhalation on trimethyltin-induced neurotoxicity and cognitive impairment in the C57BL/6 mice model. International Journal of Molecular Sciences.

[b13-tlsr-34-3-165] Kawagishi H, Shimada A, Shirai R, Okamoto K, Ojima F, Sakamoto H, Ishiguro Y, Furukawa S (1994). Erinacines A, B and C, strong stimulators of nerve growth factor (NGF)-synthesis, from the mycelia of *Hericium erinaceum*. Tetrahedron Letters.

[b14-tlsr-34-3-165] Kim S, Chen J, Cheng T, Gindulyte A, He J, He S, Li Q, Shoemaker BA, Thiessen PA, Yu B, Zaslavsky L, Zhang J, Bolton EE (2020). PubChem in 2021: New data content and improved web interfaces. Nucleic Acids Research.

[b15-tlsr-34-3-165] Lee EW, Shizuki K, Hosokawa S, Suzuki M, Suganuma H, Inakuma T, Li J, Ohnishi-Kameyama M, Nagata T, Furukawa S, Kawagishi H (2000). Two novel diterpenoids, erinacines H and I from the mycelia of *Hericium erinaceum.*. Bioscience, Biotechnology, and Biochemistry.

[b16-tlsr-34-3-165] Lee S, Yang M, Kim J, Kang S, Kim J, Kim JC, Jung C, Shin T, Kim SH, Moon C (2016). Trimethyltin-induced hippocampal neurodegeneration: A mechanism-based review. Brain Research Bulletin.

[b17-tlsr-34-3-165] Lee SC, Zhao ML, Hirano A, Dickson DW (1999). Inducible nitric oxide synthase immunoreactivity in the Alzheimer disease hippocampus: Association with Hirano bodies, neurofibrillary tangles, and senile plaques. Journal of Neuropathology & Experimental Neurology.

[b18-tlsr-34-3-165] Li E, Kim DH, Cai M, Lee S, Kim Y, Lim E, Ryu JH, Unterman TG, Park S (2011). Hippocampus-dependent spatial learning and memory are impaired in growth hormone-deficient spontaneous dwarf rats. Endocrine Journal.

[b19-tlsr-34-3-165] Li G, Yu K, Li F, Xu K, Li J, He S, Cao S, Tan G (2014). Anticancer potential of *Hericium erinaceus* extracts against human gastrointestinal cancers. Journal of Ethnopharmacology.

[b20-tlsr-34-3-165] Li IC, Lee LY, Tzeng TT, Chen WP, Chen YP, Shiao YJ, Chen CC (2018). Neurohealth properties of *Hericium erinaceus* mycelia enriched with erinacines. Behavioural Neurology.

[b21-tlsr-34-3-165] Ma B-J, Shen J-W, Yu H-Y, Ruan Y, Wu T-T, Zhao X (2010). Hericenones and erinacines: Stimulators of nerve growth factor (NGF) biosynthesis in *Hericium erinaceus*. Mycology.

[b22-tlsr-34-3-165] Manyagasa N, Thong-asa W (2019). The effects of p-hydroxycinnamic acid in ameliorating spatial learning and flexibility deficits in rats with chronic cerebral hypoperfusion. Sains Malaysiana.

[b23-tlsr-34-3-165] Martin EI, Ressler KJ, Binder E, Nemeroff CB (2009). The neurobiology of anxiety disorders: Brain imaging, genetics, and psychoneuroendocrinology. Psychiatric Clinics of North America.

[b24-tlsr-34-3-165] Mattila PM, Rinne JO, Helenius H, Roytta M (1999). Neuritic degeneration in the hippocampus and amygdala in Parkinson’s disease in relation to Alzheimer pathology. Acta Neuropathologica.

[b25-tlsr-34-3-165] Nagano M, Shimizu K, Kondo R, Hayashi C, Sato D, Kitagawa K, Ohnuki K (2010). Reduction of depression and anxiety by 4 weeks *Hericium erinaceus* intake. Biomedical Research.

[b26-tlsr-34-3-165] Ogita K, Nitta Y, Watanabe M, Nakatani Y, Nishiyama N, Sugiyama C, Yoneda Y (2004). In vivo activation of c-Jun N-terminal kinase signaling cascade prior to granule cell death induced by trimethyltin in the dentate gyrus of mice. Neuropharmacology.

[b27-tlsr-34-3-165] Paxinos G, Franklin K (2008). The mouse brain in stereotaxic coordinates.

[b28-tlsr-34-3-165] Sakamula R, Thong-asa W (2018). Neuroprotective effect of p-coumaric acid in mice with cerebral ischemia reperfusion injuries. Metabolic Brain Disease.

[b29-tlsr-34-3-165] Shi L, Adams MA, Long A, Carter CC, Bennett C, Sonntag WE, Nicolle MM, Robbins M, D’Agostino R, Brunso-Bechtold JK (2006). Spatial learning and memory deficits after whole-brain irradiation are associated with changes in NMDA receptor subunits in the hippocampus. Radiation Research.

[b30-tlsr-34-3-165] Silvers JM, Tokunaga S, Berry RB, White AM, Matthews DB (2003). Impairments in spatial learning and memory: Ethanol, allopregnanolone, and the hippocampus. Brain Brain Research Reviews.

[b31-tlsr-34-3-165] Somredngan S, Thong-asa W (2017). Neurological changes in vulnerable brain areas of chronic cerebral hypoperfusion mice. Annals of Neurosciences.

[b32-tlsr-34-3-165] Sze CI, Troncoso JC, Kawas C, Mouton P, Price DL, Martin LJ (1997). Loss of the presynaptic vesicle protein synaptophysin in hippocampus correlates with cognitive decline in Alzheimer disease. Journal of Neuropathology & Experimental Neurology.

[b33-tlsr-34-3-165] Thong-Asa W, Bullangpoti V (2020). Neuroprotective effects of *Tiliacora triandra* leaf extract in a mice model of cerebral ischemia reperfusion. Avicenna Journal of Phytomedicine.

[b34-tlsr-34-3-165] Thong-Asa W, Prasartsri S, Klomkleaw N, Thongwan N (2020). The neuroprotective effect of betanin in trimethyltin-induced neurodegeneration in mice. Metabolic Brain Disease.

[b35-tlsr-34-3-165] Thong-Asa W, Tumkiratiwong P, Bullangpoti V, Kongnirundonsuk K, Tilokskulchai K (2017). *Tiliacora triandra* (Colebr.) Diels leaf extract enhances spatial learning and learning flexibility, and prevents dentate gyrus neuronal damage induced by cerebral ischemia/reperfusion injury in mice. Avicenna Journal of Phytomedicine.

[b36-tlsr-34-3-165] Wang JC, Hu SH, Wang JT, Chen KS, Chia YC (2005). Hypoglycemic effect of extract of *Hericium erinaceus*. Journal of the Science of Food and Agriculture.

[b37-tlsr-34-3-165] Wang M, Li B, Wang C, Chen Y, Zuo Z (2008). The concentration-dependent induction of cell death by trimethyltin chloride in rat liver epithelial IAR20 cells. Toxicology in Vitro.

[b38-tlsr-34-3-165] Win-Shwe TT, Yamamoto S, Fujitani Y, Hirano S, Fujimaki H (2008). Spatial learning and memory function-related gene expression in the hippocampus of mouse exposed to nanoparticle-rich diesel exhaust. Neurotoxicology.

[b39-tlsr-34-3-165] Yoneyama M, Seko K, Kawada K, Sugiyama C, Ogita K (2009). High susceptibility of cortical neural progenitor cells to trimethyltin toxicity: Involvement of both caspases and calpain in cell death. Neurochemistry International.

